# Supplementary material for: The incidence, maternal, fetal and neonatal consequences of single intrauterine fetal death in monochorionic twins: A prospective observational UKOSS study
Source: PLoS One. 2020 Sep 21;15(9):e0239477. doi: 10.1371/journal.pone.0239477 (PMC7505445; doi:10.1371/journal.pone.0239477)
Supplement: Attachment — Submitted filename: Response to reviewers.doc [file pone.0239477.s001.doc]

**College of Medical and Dental Sciences**

Institute of Applied Health Research

Dr R.Katie Morris

University of Birmingham

Birmingham Women’s Hospital

Birmingham

B15 2TG

Tel:	+44 (0)121 626 4535

E-mail:	r.k.morris@bham.ac.uk

The Editor

*PLOS One*

September 2^nd^ 2020

Dear Sir/Madam,

**RE: The incidence, maternal, fetal and neonatal consequences of single intrauterine fetal death in monochorionic twins: a prospective observational UKOSS study**

Thank you for your kind consideration of our manuscript and for the helpful comments from the reviewers. We confirm that we have made the formatting changes to conform to the journal style (untracked). We respond to the reviewers’ and Editors’ comments below.

1. **Data Sharing**

Data cannot be shared publicly because of confidentiality issues and potential identifiability of sensitive data as identified within the Research Ethics Committee application/approval. Requests to access the data can be made by contacting the National Perinatal Epidemiology Unit data access committee via [general@npeu.ox.ac.uk](mailto:general@npeu.ox.ac.uk).

2. **Ethics statement**

This has been added to the methods section.

**3. Reviewer 1 comments**

*Of those with TTTS, what were the details for mean gestation etc?*

This data has been added to the antenatal intervention section allowing a comparison with the median for spontaneous sIUFD.

*Were there any noticeable differences in the deaths between those that did and did not have intervention?*

For the pregnancies with TTTS (n=7) that did not undergo intervention these constituted a different group to those that underwent intervention for the following reasons: late diagnosis (third trimester), intrapartum acute TTTS, TTTS complicated with sIUGR, stage 5 at diagnosis and one case of Quintero stage 1. Thus comparison between the two groups (TTTS intervention v TTTS no-intervention) would not be valid. Due to the small numbers within this group it is not possible for us to list the information as provided here due to single cases allowing identification. We have added a sentence to the section on antenatal intervention to explain this.

*Was there anything to add about gestations at which fetal therapy was performed and Staging of TTS at time of intervention.*

Quintero stage was not reliable recorded across the 38 TTTS cases and thus no further analysis on this was performed. For gestation of fetal therapy, apart from the cases where antenatal intervention was not performed due to Quintero stage 5 at diagnosis or third trimester diagnosis the gestation of diagnosis and timing of fetal therapy were very similar. To ensure that the focus of this paper is not around the management of TTTS nor the outcomes for management of this condition – for which there are much bigger cohorts published and non-selected cohorts – we have elected not to discuss this further.

*For the sepsis cases what was the timeframe relationship to the IUFD and did this result in second twin demise?*

We have added the explanation that the 2 NND were related to preterm premature rupture of membranes after intervention leading to prematurity. These same 2 NND were for the mothers that had sepsis. We have added that premature rupture of membranes was related to the maternal sepsis and ITU admission. To ensure that cases are not identifiable we have not linked the neonatal to the maternal outcomes.

*What were the causes of the two NND in co-twins (from certificates)?*

These were listed as extreme prematurity as described in the section “Surviving Co-Twin Outcomes”.

*Is there anything to explain why only 36 have brain imaging antenatally?*

We are not able to determine why this was only recorded for 36 of the cases. We believe that it may be due to the way this question was listed as “Antenatal brain imaging” and thus some centres/reporters interpreted this as specialist (fetal medicine) imaging only.

*Was TOP offered for the 7 with findings that were abnormal antenatally? If so, how many accepted TOP?*

We did not ask whether TOP had been offered, as this would be routine practice within the UK to do so for a significant pathological finding antenatally.

*Similarly, is there any indication of why only 9/62 in NNU have brain imaging?*

We believe that underreporting may play a part in this funding due to many of the UKOSS reporters not being neonatologists. This relates to the point below and response.

*I think the lack of provision of CNS imaging and CNS follow-up for surviving twins is the most striking finding of the study and it would be good to add these details if they are available and also to discuss how the authors would propose to address this variation in provision across the UK.*

Thank you, we agree with this entirely and believe that this is one of the most important messages to come from this work. We reported that the commonest aetiology of single twin demise was compilations of twin to twin transfusion syndrome and for co-twin demise complications following intervention. We highlight that monochorionic twin pregnancies with single IUD are complex and require specialist care. Further research is required regarding optimal gestation at delivery of the surviving co-twin, preterm birth prevention, and classifying the cause of death in twin pregnancies. Awareness of the importance of CNS imaging, and follow-up, needs improvement. This is highlighted in the conclusion within the abstract as well as the manuscript.

We make two suggestions within the manuscript:

1. That there is a need to strengthen professional guidance and practice amongst both obstetricians and neonatal paediatricians. We cite the most recent RCOG guidance for management of monochorionic twin pregnancies from 2016 which only say that MRI may be performed following single twin demise with no discussion of imaging postnatally. The authors are not aware of any nationally produced neonatal guidance.

2. There is a need for a purposely designed prospective study with antenatal and postnatal imaging and long-term follow-up for single twin demise.

We look forward to hearing from you in the near future.

Yours sincerely, on behalf of all of the authors,

Dr R.Katie Morris

Reader Maternal Fetal Health
